# Regular dental check-ups could be of benefit for patients receiving intravenous bisphosphonates. Regarding ‘risks and benefits of bisphosphonates’

**DOI:** 10.1038/sj.bjc.6604550

**Published:** 2008-08-05

**Authors:** A Kyrgidis, S Triaridis

**Affiliations:** 1 Department of Oral and Maxillofacial Surgery, Theagenion Cancer Hospital, Thessaloniki, Greece; 2 1st University Department of Otolaryngology, Aristotle University, AHEPA Hospital, Thessaloniki, Greece

**Sir**,

We read with considerable interest the review by RE Coleman (2008) discussing the latest clinical aspects of bisphosphonates (BP) use and their side effects.

Coleman reports that 75% of BP-related osteonecrosis of the jaw (ONJ) occurs following a dental extraction or jaw surgery. This might be true, however, in a case–control study that we recently published, in which the percentage of breast cancer patients under zolendronic acid (ZA) treatment who developed ONJ after having experienced tooth extraction was found to be 50% (Kyrgidis *et al*, 2008). In this study, patients who experienced tooth extraction were reported to have an at least 16 times higher risk of developing ONJ. Nonetheless, tooth extraction and alveolar surgery may not be the only important clinical factor for ONJ development. In our study, we also report the use of dentures to account for an almost five-fold higher risk for ONJ development. A recent paper (Landesberg *et al*, 2008) provides evidence that the oral mucosa can actually be defective. We hypothesised that potentially ill-fit dentures could cause breaches to the defective underlying mucosa, thereby providing an entry point to the bone for oral flora (Kyrgidis and Vahtsevanos, 2008; Kyrgidis *et al*, 2008).

Coleman notes that the reported frequency of ONJ varies considerably between <1 and >10% among different populations. This fact has also been emphasised by other authors, especially in regard to the Greek and US population (Raje *et al*, 2008). We have suggested that it could be an issue of differential susceptibility to ONJ in the Greek population (Kyrgidis and Andreadis, 2008), although careful pretreatment, dental assessment and avoidance of invasive dental procedures, as suggested by the author (Coleman, 2008), could partly account for this difference. The latter hypothesis is supported by the evidence we provide (Kyrgidis *et al*, 2008) that tooth extractions may amplify the risk for ONJ development.

In accordance with Coleman, we believe that prospective research is definitely required to assess potential risk factors, effect modifiers and confounders. In this regard, we currently carry out a prospective cohort study with breast, prostate cancer and multiple myeloma patients receiving BP treatment (ZA and ibandronate). In this study, the type, dose and duration of treatment is monitored. Results will be published when the study is concluded (Kyrgidis *et al*, 2008).

Coleman has suggested that until solid evidence is available, patients receiving BP should have regular inspection of the mouth, undergo dental check-ups every 6–12 months and avoid invasive dental surgery unless no alternative is available (Coleman, 2008). Our study provides substantial evidence supporting that suggestion, at the same time raising the current ASCO level of evidence linking ONJ to tooth extractions from V to III. We further suggest, on the basis of evidence (Kyrgidis *et al*, 2008), that additional dental and medical attention should be paid to BP patients using removable dentition to avoid mucosal breaches caused by dentures.
